# Di-*tert*-butyl (1,1′-binaphthyl-2,2′-di­oxy)diacetate

**DOI:** 10.1107/S1600536809010836

**Published:** 2009-03-28

**Authors:** Asra Mustafa, Muhammad Raza Shah, Maimoona Khatoon, Seik Weng Ng

**Affiliations:** a HEJ Research Institute of Chemistry, International Center for Chemical and Biological Sciences, University of Karachi, Karachi 75270, Pakistan; b Department of Chemistry, University of Malaya, 50603 Kuala Lumpur, Malaysia

## Abstract

In the crystal structure of the title compound, C_32_H_34_O_6_, the mol­ecule is located on a twofold rotation axis. The two naphthyl fused-ring systems are aligned at 72.6 (1)°. Weak intermolecular C—H⋯O hydrogen bonding is present in the crystal structure.

## Related literature

For the crystal structure of the parent carboxylic acid, see: Wu *et al.* (2007 ▶).
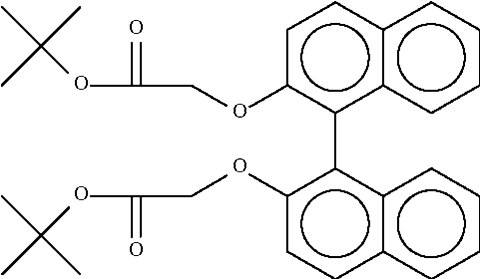


## Experimental

### 

#### Crystal data

C_32_H_34_O_6_
*M* *_r_* = 514.59
Monoclinic, 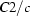

*a* = 18.7604 (3) Å
*b* = 14.3204 (3) Å
*c* = 10.9997 (2) Å
β = 110.144 (1)°
*V* = 2774.37 (9) Å^3^
*Z* = 4
Mo *K*α radiation
μ = 0.08 mm^−1^
*T* = 133 K
0.30 × 0.15 × 0.10 mm

#### Data collection

Bruker SMART APEX diffractometer
Absorption correction: none
12968 measured reflections
3198 independent reflections
2514 reflections with *I* > 2σ(*I*)
*R*_int_ = 0.035

#### Refinement

*R*[*F* ^2^ > 2σ(*F* ^2^)] = 0.043
*wR*(*F* ^2^) = 0.121
*S* = 1.01
3198 reflections
175 parameters
H-atom parameters constrained
Δρ_max_ = 0.30 e Å^−3^
Δρ_min_ = −0.20 e Å^−3^

### 

Data collection: *APEX2* (Bruker, 2008 ▶); cell refinement: *SAINT* (Bruker, 2008 ▶); data reduction: *SAINT*; program(s) used to solve structure: *SHELXS97* (Sheldrick, 2008 ▶); program(s) used to refine structure: *SHELXL97* (Sheldrick, 2008 ▶); molecular graphics: *X-SEED* (Barbour, 2001 ▶); software used to prepare material for publication: *publCIF* (Westrip, 2009 ▶).

## Supplementary Material

Crystal structure: contains datablocks global, I. DOI: 10.1107/S1600536809010836/xu2499sup1.cif

Structure factors: contains datablocks I. DOI: 10.1107/S1600536809010836/xu2499Isup2.hkl

Additional supplementary materials:  crystallographic information; 3D view; checkCIF report

## Figures and Tables

**Table 1:** Hydrogen-bond geometry (Å, °)

*D*—H⋯*A*	*D*—H	H⋯*A*	*D*⋯*A*	*D*—H⋯*A*
C9—H9⋯O2^i^	0.95	2.38	3.226 (2)	149

Symmetry code: (i) 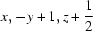.

**Fig. 1.**
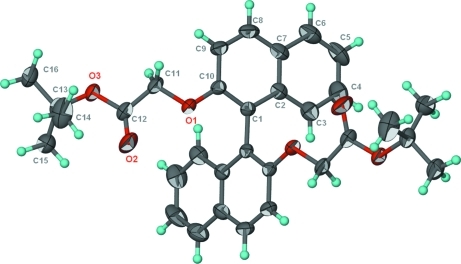
Thermal ellipsoid plot (Barbour, 2001) plot of C32H34O6 at the 70% probability level; hydrogen atoms are drawn as spheres of arbitrary radius.

## supplementary crystallographic information

### Experimental

Potassium carbonate (0.97 g, 7 mmol) and 1,1'-binaphthyl-2,2'-diol (0.57 mg, 2 mmol) in acetone (20 ml) were stirred for 15 minutes. *tert*-Butyl 2-bromoacetate (1.95 g, 10 mmol) was added and the mixture was stirred at 323 K for 2 h. The solvent was removed and the residue was dissolved in a mixture of water (50 ml) and dichloromethane (50 ml). The two phases were separated and the aqueous layer was extracted with dichloromethane. The combined organic phases were dried and the solvent evaporated. The residue was dissolved recrysstallized from dichloromethane (0.82 mg, 80% yield).

### Refinement

Carbon-bound H-atoms were placed in calculated positions (C—H 0.93 to 0.99 Å) and were included in the refinement in the riding model approximation, with *U*(H) set to 1.2 to 1.5*U*(C).

### Crystal data

**Table:**

C_32_H_34_O_6_	*F*(000) = 1096
*M**_r_* = 514.59	*D*_x_ = 1.232 Mg m^−^^3^
Monoclinic, *C*2/*c*	Mo *K*α radiation, λ = 0.71073 Å
Hall symbol: -C 2yc	Cell parameters from 3672 reflections
*a* = 18.7604 (3) Å	θ = 2.3–28.2°
*b* = 14.3204 (3) Å	µ = 0.08 mm^−^^1^
*c* = 10.9997 (2) Å	*T* = 133 K
β = 110.144 (1)°	Block, colorless
*V* = 2774.37 (9) Å^3^	0.30 × 0.15 × 0.10 mm
*Z* = 4	

### Data collection

**Table:**

Bruker SMART APEX diffractometer	2514 reflections with *I* > 2σ(*I*)
Radiation source: fine-focus sealed tube	*R*_int_ = 0.035
graphite	θ_max_ = 27.5°, θ_min_ = 1.8°
ω scans	*h* = −24→24
12968 measured reflections	*k* = −18→18
3198 independent reflections	*l* = −14→14

### Refinement

**Table:**

Refinement on *F*^2^	Primary atom site location: structure-invariant direct methods
Least-squares matrix: full	Secondary atom site location: difference Fourier map
*R*[*F*^2^ > 2σ(*F*^2^)] = 0.043	Hydrogen site location: inferred from neighbouring sites
*wR*(*F*^2^) = 0.121	H-atom parameters constrained
*S* = 1.01	*w* = 1/[σ^2^(*F*_o_^2^) + (0.0548*P*)^2^ + 1.9389*P*] where *P* = (*F*_o_^2^ + 2*F*_c_^2^)/3
3198 reflections	(Δ/σ)_max_ = 0.001
175 parameters	Δρ_max_ = 0.30 e Å^−^^3^
0 restraints	Δρ_min_ = −0.20 e Å^−^^3^

### Fractional atomic coordinates and isotropic or equivalent isotropic displacement parameters (Å^2^)

**Table:**

	*x*	*y*	*z*	*U*_iso_*/*U*_eq_	
O1	0.38962 (5)	0.53557 (7)	0.23963 (9)	0.0278 (2)	
O2	0.28361 (6)	0.45195 (10)	0.03634 (11)	0.0531 (4)	
O3	0.19998 (5)	0.45848 (7)	0.14254 (9)	0.0292 (2)	
C1	0.49788 (7)	0.63074 (9)	0.31656 (11)	0.0219 (3)	
C2	0.55167 (7)	0.68166 (9)	0.41843 (12)	0.0255 (3)	
C3	0.61052 (8)	0.73495 (10)	0.39965 (14)	0.0326 (3)	
H3	0.6162	0.7352	0.3171	0.039*	
C4	0.65937 (10)	0.78615 (12)	0.49836 (16)	0.0441 (4)	
H4	0.6980	0.8223	0.4833	0.053*	
C5	0.65281 (11)	0.78558 (13)	0.62268 (16)	0.0503 (5)	
H5	0.6869	0.8213	0.6908	0.060*	
C6	0.59820 (10)	0.73436 (12)	0.64444 (15)	0.0437 (4)	
H6	0.5948	0.7336	0.7286	0.052*	
C7	0.54571 (8)	0.68154 (10)	0.54431 (13)	0.0314 (3)	
C8	0.48693 (8)	0.62974 (12)	0.56391 (13)	0.0350 (3)	
H8	0.4833	0.6283	0.6479	0.042*	
C9	0.43514 (8)	0.58162 (11)	0.46649 (13)	0.0310 (3)	
H9	0.3958	0.5474	0.4824	0.037*	
C10	0.44042 (7)	0.58301 (9)	0.34153 (12)	0.0242 (3)	
C11	0.32140 (7)	0.50678 (11)	0.25672 (13)	0.0291 (3)	
H11A	0.2979	0.5603	0.2857	0.035*	
H11B	0.3324	0.4575	0.3238	0.035*	
C12	0.26778 (8)	0.46984 (10)	0.13024 (14)	0.0304 (3)	
C13	0.13800 (8)	0.40721 (10)	0.04369 (14)	0.0315 (3)	
C14	0.16385 (11)	0.30839 (13)	0.0342 (2)	0.0556 (5)	
H14A	0.1856	0.2817	0.1213	0.083*	
H14B	0.2023	0.3087	−0.0075	0.083*	
H14C	0.1204	0.2706	−0.0173	0.083*	
C15	0.11375 (9)	0.45879 (14)	−0.08469 (15)	0.0439 (4)	
H15A	0.1028	0.5241	−0.0710	0.066*	
H15B	0.0681	0.4294	−0.1451	0.066*	
H15C	0.1547	0.4562	−0.1208	0.066*	
C16	0.07523 (8)	0.40965 (12)	0.10104 (15)	0.0394 (4)	
H16A	0.0930	0.3799	0.1865	0.059*	
H16B	0.0308	0.3760	0.0440	0.059*	
H16C	0.0613	0.4746	0.1097	0.059*	

### Atomic displacement parameters (Å^2^)

**Table:**

	*U*^11^	*U*^22^	*U*^33^	*U*^12^	*U*^13^	*U*^23^
O1	0.0218 (5)	0.0396 (6)	0.0254 (5)	−0.0038 (4)	0.0123 (4)	−0.0006 (4)
O2	0.0342 (6)	0.0883 (10)	0.0461 (7)	−0.0154 (6)	0.0255 (5)	−0.0245 (6)
O3	0.0220 (5)	0.0364 (5)	0.0321 (5)	−0.0025 (4)	0.0131 (4)	−0.0021 (4)
C1	0.0220 (6)	0.0255 (6)	0.0194 (6)	0.0059 (5)	0.0088 (5)	0.0020 (5)
C2	0.0265 (7)	0.0256 (6)	0.0225 (6)	0.0064 (5)	0.0062 (5)	−0.0007 (5)
C3	0.0345 (7)	0.0302 (7)	0.0292 (7)	−0.0021 (6)	0.0061 (6)	−0.0016 (6)
C4	0.0416 (9)	0.0373 (8)	0.0456 (9)	−0.0091 (7)	0.0050 (7)	−0.0067 (7)
C5	0.0545 (10)	0.0477 (10)	0.0339 (8)	−0.0049 (8)	−0.0037 (8)	−0.0146 (7)
C6	0.0508 (10)	0.0487 (9)	0.0261 (7)	0.0031 (8)	0.0060 (7)	−0.0089 (7)
C7	0.0340 (7)	0.0357 (7)	0.0214 (6)	0.0105 (6)	0.0054 (6)	−0.0029 (5)
C8	0.0367 (8)	0.0513 (9)	0.0199 (6)	0.0129 (7)	0.0134 (6)	0.0035 (6)
C9	0.0282 (7)	0.0447 (8)	0.0241 (7)	0.0072 (6)	0.0142 (6)	0.0064 (6)
C10	0.0218 (6)	0.0310 (7)	0.0213 (6)	0.0062 (5)	0.0094 (5)	0.0035 (5)
C11	0.0223 (6)	0.0385 (8)	0.0310 (7)	0.0016 (5)	0.0150 (6)	0.0050 (6)
C12	0.0249 (7)	0.0345 (7)	0.0359 (7)	−0.0004 (6)	0.0157 (6)	−0.0007 (6)
C13	0.0240 (6)	0.0332 (7)	0.0377 (8)	−0.0051 (6)	0.0110 (6)	−0.0043 (6)
C14	0.0443 (10)	0.0366 (9)	0.0848 (14)	−0.0032 (8)	0.0207 (10)	−0.0131 (9)
C15	0.0359 (8)	0.0621 (11)	0.0335 (8)	−0.0065 (8)	0.0118 (7)	0.0006 (7)
C16	0.0262 (7)	0.0516 (10)	0.0419 (9)	−0.0063 (7)	0.0138 (6)	0.0024 (7)

### Geometric parameters (Å, °)

**Table:**

O1—C10	1.3749 (16)	C8—C9	1.360 (2)
O1—C11	1.4174 (15)	C8—H8	0.9500
O2—C12	1.1966 (17)	C9—C10	1.4118 (17)
O3—C12	1.3347 (15)	C9—H9	0.9500
O3—C13	1.4842 (16)	C11—C12	1.504 (2)
C1—C10	1.3815 (17)	C11—H11A	0.9900
C1—C2	1.4237 (18)	C11—H11B	0.9900
C1—C1^i^	1.494 (2)	C13—C14	1.511 (2)
C2—C3	1.415 (2)	C13—C16	1.5158 (19)
C2—C7	1.4270 (18)	C13—C15	1.518 (2)
C3—C4	1.368 (2)	C14—H14A	0.9800
C3—H3	0.9500	C14—H14B	0.9800
C4—C5	1.415 (2)	C14—H14C	0.9800
C4—H4	0.9500	C15—H15A	0.9800
C5—C6	1.347 (3)	C15—H15B	0.9800
C5—H5	0.9500	C15—H15C	0.9800
C6—C7	1.417 (2)	C16—H16A	0.9800
C6—H6	0.9500	C16—H16B	0.9800
C7—C8	1.406 (2)	C16—H16C	0.9800
			
C10—O1—C11	116.08 (10)	O1—C11—H11A	109.9
C12—O3—C13	121.30 (11)	C12—C11—H11A	109.9
C10—C1—C2	119.15 (11)	O1—C11—H11B	109.9
C10—C1—C1^i^	120.24 (12)	C12—C11—H11B	109.9
C2—C1—C1^i^	120.60 (12)	H11A—C11—H11B	108.3
C3—C2—C1	122.59 (12)	O2—C12—O3	126.15 (14)
C3—C2—C7	117.92 (13)	O2—C12—C11	126.00 (13)
C1—C2—C7	119.47 (12)	O3—C12—C11	107.83 (11)
C4—C3—C2	121.09 (14)	O3—C13—C14	108.95 (12)
C4—C3—H3	119.5	O3—C13—C16	102.04 (11)
C2—C3—H3	119.5	C14—C13—C16	111.35 (14)
C3—C4—C5	120.45 (16)	O3—C13—C15	110.43 (12)
C3—C4—H4	119.8	C14—C13—C15	113.09 (15)
C5—C4—H4	119.8	C16—C13—C15	110.43 (13)
C6—C5—C4	119.97 (15)	C13—C14—H14A	109.5
C6—C5—H5	120.0	C13—C14—H14B	109.5
C4—C5—H5	120.0	H14A—C14—H14B	109.5
C5—C6—C7	121.34 (15)	C13—C14—H14C	109.5
C5—C6—H6	119.3	H14A—C14—H14C	109.5
C7—C6—H6	119.3	H14B—C14—H14C	109.5
C8—C7—C6	122.29 (13)	C13—C15—H15A	109.5
C8—C7—C2	118.50 (13)	C13—C15—H15B	109.5
C6—C7—C2	119.20 (14)	H15A—C15—H15B	109.5
C9—C8—C7	122.11 (12)	C13—C15—H15C	109.5
C9—C8—H8	118.9	H15A—C15—H15C	109.5
C7—C8—H8	118.9	H15B—C15—H15C	109.5
C8—C9—C10	119.30 (13)	C13—C16—H16A	109.5
C8—C9—H9	120.3	C13—C16—H16B	109.5
C10—C9—H9	120.3	H16A—C16—H16B	109.5
O1—C10—C1	116.85 (10)	C13—C16—H16C	109.5
O1—C10—C9	121.70 (12)	H16A—C16—H16C	109.5
C1—C10—C9	121.44 (12)	H16B—C16—H16C	109.5
O1—C11—C12	109.05 (10)		
			
C10—C1—C2—C3	−177.72 (12)	C7—C8—C9—C10	0.3 (2)
C1^i^—C1—C2—C3	0.71 (18)	C11—O1—C10—C1	−164.92 (11)
C10—C1—C2—C7	0.70 (18)	C11—O1—C10—C9	16.29 (18)
C1^i^—C1—C2—C7	179.14 (11)	C2—C1—C10—O1	179.49 (11)
C1—C2—C3—C4	177.29 (13)	C1^i^—C1—C10—O1	1.05 (16)
C7—C2—C3—C4	−1.2 (2)	C2—C1—C10—C9	−1.72 (19)
C2—C3—C4—C5	1.1 (2)	C1^i^—C1—C10—C9	179.83 (11)
C3—C4—C5—C6	0.0 (3)	C8—C9—C10—O1	179.96 (12)
C4—C5—C6—C7	−1.0 (3)	C8—C9—C10—C1	1.2 (2)
C5—C6—C7—C8	−178.13 (16)	C10—O1—C11—C12	171.26 (11)
C5—C6—C7—C2	0.9 (2)	C13—O3—C12—O2	9.7 (2)
C3—C2—C7—C8	179.26 (13)	C13—O3—C12—C11	−168.85 (11)
C1—C2—C7—C8	0.76 (19)	O1—C11—C12—O2	12.1 (2)
C3—C2—C7—C6	0.2 (2)	O1—C11—C12—O3	−169.43 (11)
C1—C2—C7—C6	−178.34 (13)	C12—O3—C13—C14	60.23 (17)
C6—C7—C8—C9	177.77 (14)	C12—O3—C13—C16	178.05 (13)
C2—C7—C8—C9	−1.3 (2)	C12—O3—C13—C15	−64.54 (16)

Symmetry codes: (i) −*x*+1, *y*, −*z*+1/2.

### Hydrogen-bond geometry (Å, °)

**Table:**

*D*—H···*A*	*D*—H	H···*A*	*D*···*A*	*D*—H···*A*
C9—H9···O2^ii^	0.95	2.38	3.226 (2)	149

Symmetry codes: (ii) *x*, −*y*+1, *z*+1/2.
